# Molecular Profiling of Peanut under Raw, Roasting, and Autoclaving Conditions Using High-Resolution Magic Angle Spinning and Solution ^1^H NMR Spectroscopy

**DOI:** 10.3390/molecules29010162

**Published:** 2023-12-27

**Authors:** Casey G. Cohen, Bruce D. Mazer, Bertrand J. Jean-Claude

**Affiliations:** 1 The Research Institute of the McGill University Health Centre, Department of Medicine, Faculty of Medicine and Health Sciences, McGill University, Montreal, QC H4A 3J1, Canada; 2 Department of Pediatrics, Faculty of Medicine and Health Sciences, McGill University, Montreal, QC H4A 3J1, Canada

**Keywords:** peanut, food processing, autoclaving, nuclear magnetic resonance, molecular profiling

## Abstract

Higher rates of peanut allergy have been observed in countries that commonly roast peanuts prior to consumption. Despite the importance of understanding the role of thermal processing in allergy and on peanut composition, studies toward generating signatures that identify molecular contents following processing are scant. Here, we identified spectral signatures to track changes and differences in the molecular composition of peanuts under raw, roasted, and high-pressure and high-temperature autoclaved conditions. We analyzed both the solid flesh of the seed and solutions derived from soaking peanuts using High-Resolution Magic Angle Spinning (HR-MAS) and solution ^1^H Nuclear Magnetic Resonance (NMR) spectroscopy, respectively. The NMR spectra of intact peanuts revealed triglycerides as the dominant species, assigned on the basis of multiplets at 4.1 and 4.3 ppm, and corresponding defatted flours revealed the presence of sugars. Sucrose assigned based on a doublet at 5.4 ppm (anomeric proton), and triglycerides were the most abundant small molecules observed, with little variation between conditions. Soaked peanut solutions were devoid of lipids, and their resulting spectra matched the profiles of defatted peanuts. Spectral signatures resulting from autoclaving differed strikingly between those from raw and roasted peanuts, with considerable line-broadening in regions corresponding to proteins and amino-acid side chains, from 0.5 to 2.0 ppm and 6.5 to 8.5 ppm. Taken together, by using complementary NMR methods to obtain a fingerprint of the molecular components in peanuts, we demonstrated that autoclaving led to a distinct composition, likely resulting from the hydrolytic cleavage of proteins, the most important molecule of the allergic reaction.

## 1. Introduction

Peanuts, *Arachis hypogaea*, are an important legume crop worldwide, serving as an excellent and affordable source of protein, fat, and several vitamins and minerals [1]. Peanuts are frequently processed prior to consumption. In North America and Western Europe, peanuts are commonly dry roasted, particularly in the preparation of peanut butter. In Asia, it is common for peanuts to be boiled in briny water. Importantly, for a small but increasing percentage of the population in Western societies, an allergy to peanuts is a condition with a high risk of severe allergic reactions, requiring constant vigilance to avoid any accidental exposure [2]. The allergic reaction to peanuts is characterized by immunoglobulin E (IgE) antibodies binding and cross-linking to specific protein molecules known as allergens [3,4]. Most peanut allergens play the role of seed storage proteins and are tightly organized into a unique, complex matrix of membrane-bound organelles, referred to as protein bodies [5,6].

Several studies suggest significant changes in the allergenic properties of the peanut following thermal processing [7,8,9]. Protein glycation at a high temperature is proposed to enhance antibody binding to allergens, primarily resulting from the Maillard reaction, an addition of amines on reducing sugars that leads to Schiff bases and other intermediates before reacting to form many different advanced glycation end products (AGEs) [10,11]. Maleki et al. (2000) simulated roasting and the Maillard reaction by incubating allergen extracts in the presence of glucose [12]. They found that the glycated peanut proteins bound IgE in sera from peanut-allergic patients approximately 90-fold more when compared to raw peanuts, though evidence of increased IgE binding to commercially roasted peanut proteins is lacking. Despite the ongoing debate over whether roasting and the Maillard reaction play a role in enhancing the allergic reaction to foods [13], the effects of other processing methods on allergenicity have shown more promise in reducing allergenicity. Cabanillas et al. (2012 & 2015) demonstrated that high-pressure and high-temperature autoclaving of peanuts resulted in decreased major allergen detection and alterations in the protein secondary structure [14,15]. More recently, the same group found that the combination of thermal, pressure, and enzymatic processing of peanuts was efficient at considerably reducing IgE binding in allergic patient sera via ELISA, immunoblot, and skin prick test [16]. Similar results were observed with other foods, such as the tree nuts pistachio and cashew [17,18]. Our laboratory recently showed that autoclaving significantly hydrolyzes peanut proteins, thereby decreasing IgE binding to peanut allergens when compared to raw or roasted peanuts [19].

Despite substantial evidence of the effect of thermal processing on the legume, scant attention has been paid to signatures associated with the physical and chemical changes that characterize peanut composition as a function of allergenicity. We surmised that defining small molecule and protein profiles of the peanut under different conditions could help establish an indirect signature specific for raw and/or thermally processed peanuts, particularly roasted and autoclaved ones. Such signatures could also be developed as a method to distinguish and label differentially processed peanuts based on allergenicity. The chemical composition of peanuts consists primarily of lipids, proteins, and small molecules, such as amino acids, reducing and non-reducing sugars [1,20,21]. At high temperatures, a myriad of chemical reactions occur, leading to a complex, multifarious mixture of protein products. Accordingly, monitoring changes in the molecular profiles of intact seeds or soaked extracts from peanuts requires a technique that can permit one to capture a signature of both small molecules and proteins under different processing conditions.

Given that nuclear magnetic resonance (NMR) spectroscopy is a technique that provides detailed information about the structure, dynamics, and chemical environment of molecules through specific chemical shifts, we used ^1^H NMR spectroscopy to obtain molecular distribution profiles reflecting the various peanut compositions under raw, roasted, and autoclaving conditions. Furthermore, recent advances in solid-state NMR have led to the development of high-resolution magic angle spinning (HR-MAS) approaches that allow the analysis of intact tissues by spinning the samples at a very specific angle of 54.7° to minimize chemical shift anisotropic interactions, resulting in spectra with a very high resolution [20,21]. This makes the HR-MAS NMR technology ideal to read the molecular composition of food products in situ [22,23]. Here, we report our results from complementary NMR analyses of both intact and aqueous solutions resulting from soaking raw and thermally processed peanuts.

## 2. Results

### 2.1. Predominance of Lipids Observed through Intact Peanut Analysis

**Figure 1:**
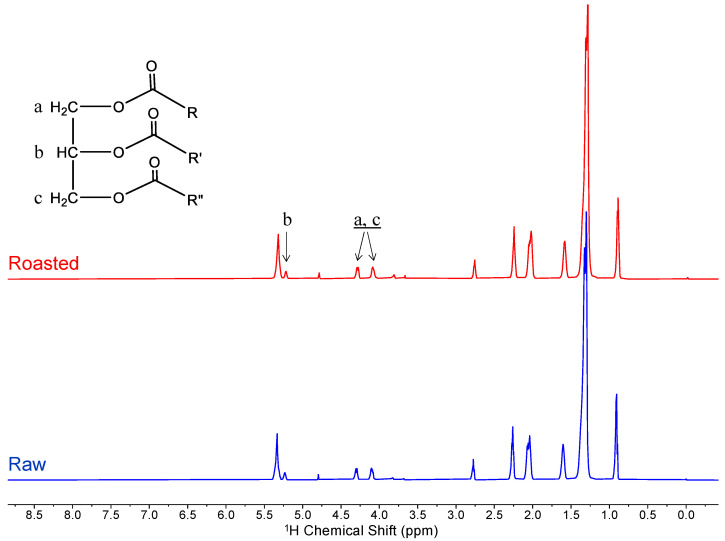
HR-MAS ^1^H NMR spectra of raw and roasted intact peanut. Shaded boxes highlight peaks corresponding to triglyceride protons, which dominate the spectra. In the general structure of the triglyceride molecule shown, R, R′, and R″ represent aliphatic or olefinic side chains.

The HR-MAS ^1^H NMR profiles demonstrated that lipids were the dominant component of raw and thermally processed peanuts in their whole, intact form (Figure 1). The lipids observed were primarily triglycerides with characteristic proton signals at 4.1 and 4.3 ppm corresponding to the pair of CH_2′_s and ones at 5.2 ppm corresponding to the central CH of the glycerol backbone. The peak at 5.3 ppm corresponds to olefinic protons, matching the reported spectrum of peanut oil [24,25,26]. Analysis of roasted peanuts did not show a noticeable difference in the lipid profile when compared to raw ones. Lipids are stable under high temperatures and therefore were not expected to degrade or react with other biomolecules in the peanut. Although lipids were the predominant signal in the spectra, when the scale was magnified, peaks could be observed from 3.5 to 4.0 ppm, corresponding to other molecules, such as sugars. This implies that the HR-MAS analysis captures signals corresponding not only to lipids but also to other small molecules in the peanut. However, due to the high proportion of lipids, peaks corresponding to other small molecules were masked in the spectra.

### 2.2. Sugars Revealed through the Analysis of Peanut Flour

**Figure 2:**
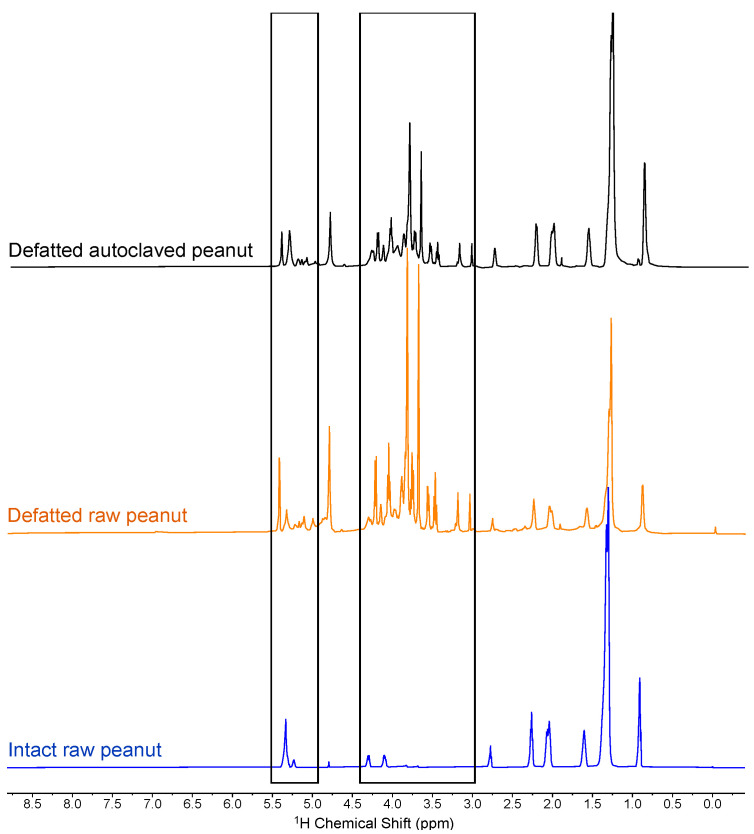
HR-MAS ^1^H NMR spectra of autoclaved peanut flour, raw peanut flour, and whole, intact raw peanut. Peanut flour was defatted with hexane. Shaded boxes highlight sugar-corresponding peaks, revealed through defatting.

To unveil signals corresponding to less abundant molecules masked by lipids, we defatted the samples through suspension and washing in hexanes. HR-MAS analysis of the resulting peanut flour showed a marked decrease in lipid-corresponding peaks, revealing signals that indicate the presence of sucrose (Figure 2). This assignment was based on the detection of a doublet at 5.4 ppm corresponding to the glucopyranose anomeric proton, as well as multiple carbohydrate-corresponding peaks from 3.0 to 4.5 ppm. This was in agreement with sucrose being the dominant sugar in peanuts, representing approximately 88% of all sugars [27]. Minor peaks at 5.2 ppm suggested the presence of glucose as well. The thermally processed spectra showed no major differences compared to raw peanuts in the HR-MAS spectra of defatted flour, and sucrose was the dominant sugar detected in each spectrum. Although defatting resulted in the resolution of peaks corresponding to sugars, signals related to triglycerides remained observable and covered the region from 0.5 to 2.5 ppm where protons of side chains of amino acids resonate.

### 2.3. Detecting Proteins through the Analysis of Soaked Peanut Solutions

**Figure 3:**
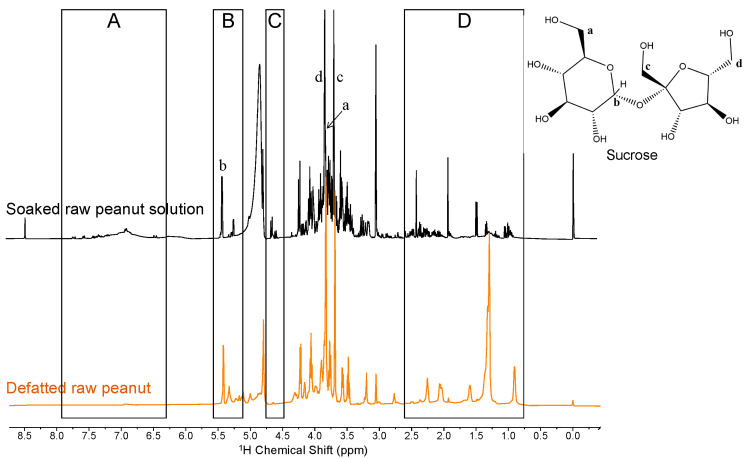
HR-MAS ^1^H NMR spectrum of defatted raw peanut flour and solution. ^1^H NMR spectrum of a peanut-soaked solution of six raw peanuts in distilled water for 24 h. Shaded boxes highlight: (**A**,**D**) regions containing protons of peptide and amino-acid side chains of proteins, uncovered via soaking the peanuts in water and (**B**,**C**) peaks corresponding to the anomeric protons of sucrose and glucose. Lowercase letters highlight select peaks corresponding to protons in the structure of sucrose.

**Figure A1:**
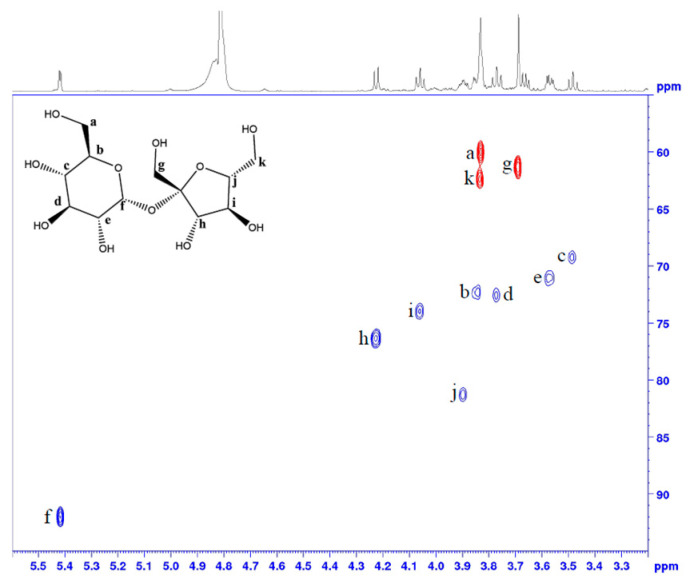
2D HSQC ^1^H–^13^C solution NMR analyses of soaked solutions derived from **raw** peanuts soaked in water for 24 h. Lowercase letter assignments highlight protons on the corresponding carbons of sucrose. CH peaks are in blue and CH_2_ peaks are in red.

**Figure A2:**
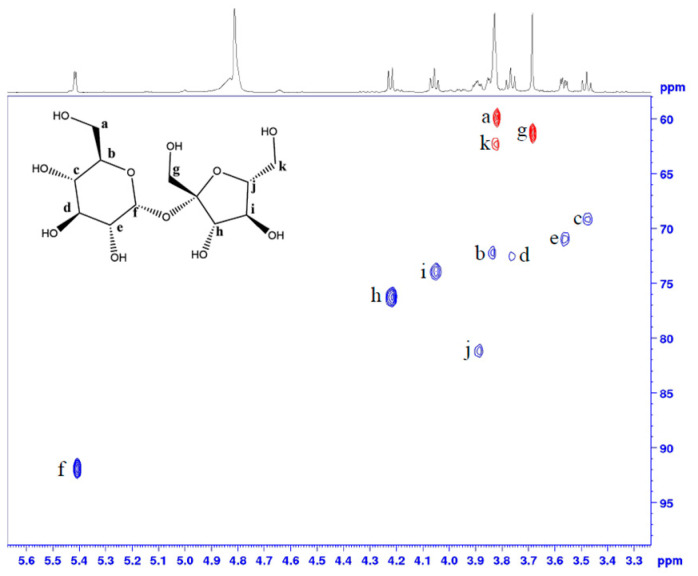
2D HSQC ^1^H–^13^C solution NMR analyses of soaked solutions derived from **roasted** peanuts soaked in water for 24 h. Lowercase letter assignments highlight protons on the corresponding carbons of sucrose. CH peaks are in blue and CH_2_ peaks are in red.

**Figure A3:**
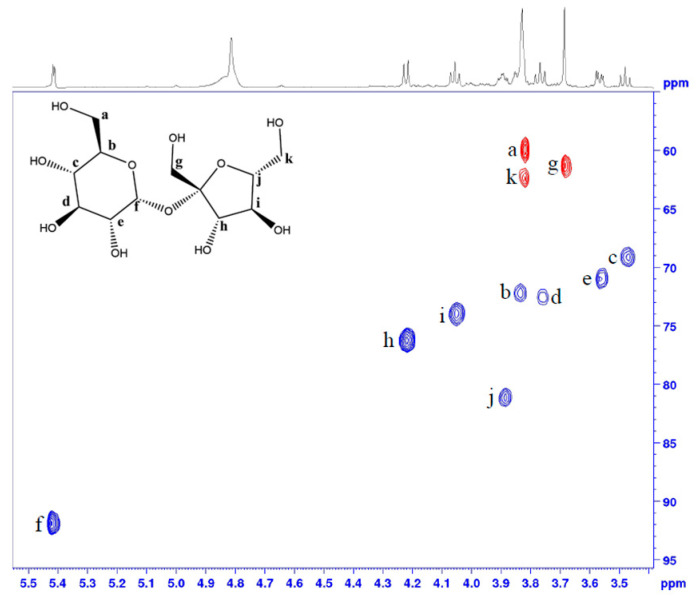
2D HSQC ^1^H–^13^C solution NMR analyses of soaked solutions derived from **autoclaved** peanuts soaked in water for 24 h. Lowercase letter assignments highlight protons on the corresponding carbons of sucrose. CH peaks are in blue and CH_2_ peaks are in red.

To obtain a full picture of water-soluble molecules in peanuts and circumvent the interference by lipids, we soaked whole raw seeds in water and analyzed the resulting solution by ^1^H NMR (Figure 3). As with the analysis of defatted peanut flour, we used the peak of the anomeric protons to assign the presence of sucrose and glucose; small doublets at 5.2 and 4.7 ppm were assigned to the α- and β-anomeric protons of glucose, respectively (Figure 3B,C). 2D HSQC NMR experiments of soaked solutions unequivocally confirmed the structure of sucrose, which was much more abundant than glucose under all conditions (Figure A1, Figure A2 and Figure A3) [28]. By contrast to defatted peanut flour, no lipids remained in the soaked solution, revealing peaks in the region from 0.5 to 2.5 ppm, which were assigned to aliphatic protons of free amino acids and protein amino-acid side chains. Likewise, peaks in the region from 6.5 to 8.5 ppm were assigned to aromatic and amide protons of free amino acids and protein side chains (Figure 3A,D).

### 2.4. Differential Signature for Autoclaved Peanut

**Figure 4:**
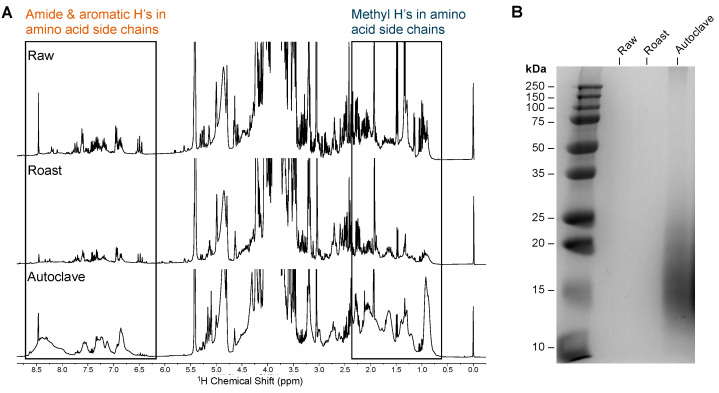
(**A**) Solution ^1^H NMR spectra of peanut-soaked solutions of six whole raw, roasted, or autoclaved peanuts in distilled water for 24 h. Shaded boxes highlight peaks corresponding to H atoms in peptide and amino-acid side chains. (**B**) Coomassie stain of proteins following SDS PAGE of the peanut-soaked solutions of each condition showing protein band distributions.

A striking difference was observed in the shape and number of signals in the autoclaved peanut spectrum when compared with the raw or roasted spectra, particularly in the regions between 0.5 to 2.5 ppm and the aromatic region from 6.5 to 8.5 ppm corresponding to protons of protein and amino-acid side chains (Figure 4A). This indicates significant molecular changes induced by the high-temperature and high-pressure environment of the autoclaving process. The observed line-broadening can perhaps be attributed to the abundance of hydrolyzed products (e.g., proteins and peptides). Remarkably, when raw peanut was subjected to roasting, no marked difference was observed in the regions assigned to protons of amino-acid side chains. Analysis of the different areas of the spectrum showed that the relative distribution of the sucrose-corresponding peaks did not change. Likewise, under autoclaving conditions, no significant changes were seen in the sucrose peaks.

To further confirm the hydrolytic degradation of proteins, an SDS-PAGE analysis was performed to assess the protein content of each soaked solution (Figure 4B). The results showed very little signal in the raw or roasted peanut-soaked solutions, in line with low levels of protein leaching out into the solution throughout soaking. However, a broad smear was observed at small molecular weights in the autoclaved peanut-soaked sample, suggesting that autoclaving leads to the release of hydrolytically cleaved products, particularly peptides ranging from approximately 12 to 25 kDa in size. This is in agreement with the broad peaks in the regions corresponding to proteins and amino acids in the NMR spectra, and perhaps a greater molecular diversity in the solution when compared to the other two conditions.

## 3. Discussion

The steady increase in peanut allergy incidence over the past three decades has catapulted the topic into intense investigation. Thermal processing of peanuts has been investigated in the context of both elucidating the mechanism of allergenicity and developing methods that can render them less allergenic. However, little attention has been paid to analytical methods to characterize these processed products with the purpose of understanding the effect of processing on allergenicity and composition. Our study specifically sought to monitor molecular differences between the overall composition of raw, roasted, and autoclaved peanuts. Indeed, we demonstrated differential changes between the three processing conditions using complementary NMR techniques, which provided a unique insight into the total molecular composition of the differentially processed peanuts.

As summarized in Table 1, HR-MAS NMR analysis of the intact legume primarily translated the lipid composition, while that of the defatted flour allowed the observation of both lipids and other components of the peanut. The overlapping of triglyceride peaks with other molecular signals hindered the interpretation of the spectra. Soaked solutions were deprived of the peak-masking effect of the triglycerides, providing an important complementary picture of the molecular composition of raw and processed peanuts, and tracking the molecular differences between the three conditions. Since protein denaturation and hydrolytic cleavage chemically lead to more water-soluble molecules, the soaked solutions proved to be the best medium to translate the distinct molecular diversity generated by autoclaving, which indicates that it is the most disruptive of the three conditions.

^1^H HR-MAS NMR presents the advantage of collecting NMR data related to the major molecules in the chemical composition of peanuts in their original matrix. The spectrum of intact peanuts largely contained lipids as the major molecular constituent, which remained unchanged following processing under the evaluated conditions. It is well established that peanuts are composed of approximately 50% lipids, largely dominated by triglycerides [29]. Importantly, following peanut defatting into flour, the ^1^H HR-MAS NMR spectra revealed water-soluble sugar molecules, mainly sucrose, with low levels of glucose, the distribution of which, as with the lipids, was not altered by thermal processing. The detection of glucose suggests that under conditions where the Maillard reaction could occur (e.g., roasting), there were sufficient levels of glucose available.

Since our study demonstrated the relative stability of lipids, sugars, and perhaps other small molecules unaffected by thermal processing of the peanut, we searched for a signature for macromolecules and proteins, which could be dissolved in soaked solutions. Indeed, in a previous report, 1D ^1^H NMR was used to assess the tertiary structure of purified seed storage peanut globulins Ara h 1 and Ara h 3, generating a fingerprint representing their overall structural and dynamic information [30]. Recombinant Ara h 6, an allergenic 2S albumin from peanuts, was evaluated via 2D HSQC NMR, and sequence-specific resonance assignments for ^1^H, ^15^N, and ^13^C were successfully identified, helping explain the particular secondary structures of the protein [31]. In the context of our study, 2D HSQC NMR was used to confirm the structure of sucrose and demonstrated that it remained present and intact in the mixture under all three conditions: raw, roasted, and autoclaved peanuts. Another use of NMR for analyzing peanut traces was reported by Schmitt et al. (2020) who discovered an isolated signal at 3.05 ppm corresponding to N-methyl-hydroxyproline in the ^1^H NMR spectrum of polar peanut extract as an indicator of peanut adulteration, which could be used to identify peanut additives and contaminants in foods [32].

The major finding of our study was that the molecular profiles of soaked solutions of autoclaved peanuts were dramatically different than those associated with raw and roasted peanuts. Evident peak broadening was observed in regions corresponding to amino acids and peptides (0.5–2.5 ppm and 6.5–8.5 ppm), suggesting the presence of multiple small molecules, perhaps resulting from protein hydrolysis. It has been shown that broad peaks around 8.3 ppm are a good indicator of proteins without ordered tertiary structures, because this region is characteristic of backbone amides in random-coil configurations [30]. Under the high-temperature and pressure conditions of autoclaving, line broadening may be due to protein and peptide entities being released into solution via the denaturation, hydrolytic cleavage, and/or breaking down of protein molecules into short peptides and amino acids [33,34]. This results in a range of molecular species and orientations, and thus broader peaks in the NMR spectrum. Moreover, this is corroborated by the fact that significant protein degradation was seen in the SDS PAGE analysis of autoclaved peanut-soaked solutions, which showed a smear corresponding to small proteins and peptides in the 10 and 25 kDa mass range.

Taken together, the data suggest that the high vapor pressure and temperature throughout autoclaving not only disrupt the tightly organized protein matrix of the peanut but favor hydrolytic cleavage, allowing the release of peptide fragments [6,35]. Roasting, on the other hand, is a dry process by which water is driven out of the peanut, potentially strengthening the protein matrix structure, which may explain the similarity of the roasted peanut spectra with the raw one. Autoclaving may be hydrolyzing the proteins to a point where epitope recognition by peanut-specific antibodies, and thus allergenicity, is greatly affected. These results are in corroboration with recent studies by our group and others, demonstrating via ELISA that autoclaving results in the lowest peanut-specific IgE binding when compared with raw or roasted peanuts [16,19,36]. Following further work, we ultimately aim to achieve a signature-defining molecular structure that correlates with decreased IgE binding, eventually leading to an indirect signature for allergenicity.

In conclusion, ^1^H HR-MAS analysis of whole peanuts provided a simple method to obtain a characteristic profile of the overall peanut composition. In parallel, the ^1^H NMR spectra of soaked extracts revealed clear, lipid-free spectra that translated the differential effect of processing via autoclaving on peanut proteins. Soaked peanut solutions, a medium in which all molecules were soluble, were more conducive to the detection of molecular changes resulting from these processes. The extensive degradation caused by autoclaving was sharply captured by NMR, indicating that it may help correlate molecular composition and structure to function. The NMR characterization may well represent an indirect signature of allergenicity through its ability to translate the extent of degradation, fragmentation, and leaching of peptides in solution. This is being developed into an effective quality control method for detecting allergens in food products (patent pending, [36]).

## 4. Materials and Methods

### 4.1. Sample Preparation

#### 4.1.1. Physical Processing

Raw, shelled peanuts were commercially purchased (Marché Victoria Orientale, Montreal, QC, Canada). Peanuts were roasted with their seed coating in a convection oven at 150 °C for 30 min or were autoclaved in a tabletop autoclave at 130 °C, 2.5 atm for 30 min. Analyses were performed in comparison with raw, unprocessed peanuts.

#### 4.1.2. Defatting Peanuts

Raw, roasted, and autoclaved peanuts (12 of each) were ground into a paste using a coffee grinder (Proctor Silex Fresh Grind^TM^ Coffee Grinder, Hamilton Beach, Belleville, ON, Canada). The paste was then suspended in hexanes, and the defatted peanut flour was collected by filtration under vacuum.

### 4.2. NMR Sample Preparation

#### 4.2.1. Solid Preparation

Small pieces (6 mg) of whole, intact peanuts or defatted peanut flour (4 mg) collected from raw, roasted, or autoclaved peanuts were loaded into a Kel-F disposable insert and subsequently placed inside a reusable 4 mm rotor.

#### 4.2.2. Solution Preparation

Six whole peanuts from each condition were placed in 10 mL of double-distilled water in a 15 mL Falcon tube. The peanuts were soaked in water at room temperature for 24 h. Three aliquots per condition (1 mL each) were evaporated under a SpeedVac (Waltham, MA, USA) at 45 °C for 1.5 h, and the resulting residue was reconstituted in 0.6 mL of double-distilled water. The three aliquots were combined to give a total volume of 1.8 mL, 450 μL of which was collected for analysis.

### 4.3. HR-MAS and Solution ^1^H NMR Spectroscopy

#### 4.3.1. HR-MAS ^1^H NMR Analysis of Solid Peanut

A total of 15 μL of 5 mM 3-(Trimethylsilyl-propionic-2,2,3,3-d_4_) acid (TSP-*d*_4_; Sigma-Aldrich, Oakville, ON, Canada) in 100% deuterium oxide (D_2_O; Sigma-Aldrich) was added to the rotor as an internal standard set to 0.0 ppm prior to the addition of the insert. Analysis was performed in a Bruker 600 MHz NMR spectrometer (Billerica, MA, USA) equipped with an advanced HR-MAS probe using the water suppression pulse sequence, *zgpr* (Bruker standard sequence). Sixty-four scans were acquired, with an acquisition time of 0.97 s and a spectral width of 8.4 kHz.

#### 4.3.2. Solution ^1^H NMR Analysis of Peanut-Soaked Solutions

Solution ^1^H NMR spectra were run on a Bruker 400 MHz NMR spectrometer for analysis using the *zgpr* water suppression pulse sequence. Thirty-two scans were acquired, with an acquisition time of 3 s and a spectral width of 12 kHz. The ^1^H chemical shifts were internally referenced by adding 0.5 mM of TSP-*d*_4_ set to 0.0 ppm. 2D HSQC NMR analyses (^1^H–^13^C) were performed on a Bruker 600 MHz NMR spectrometer.

### 4.4. SDS PAGE Analysis

Raw, roasted, and autoclaved peanut-soaked solutions were adjusted to protein concentrations of 0.5 mg/mL (determined by Bradford assay) and separated by sodium dodecyl-sulfate polyacrylamide gel electrophoresis (SDS-PAGE) under reducing conditions (2.5% β-mercaptoethanol). Protein gels were stained with 0.1% Coomassie Brilliant blue to visualize protein bands.

## 5. Conclusions

Our results demonstrate that NMR spectroscopy can be used to monitor changes in protein composition throughout peanut processing in order to develop characteristic signatures. Moreover, autoclaving peanuts, but not roasting them, resulted in the widespread cleavage of proteins into peptide fragments. Studies are ongoing to further investigate the signatures associated with and the clinical uses of autoclaved peanuts.

## 6. Patents

Patent applications were filed in the United States of America (US20220125916A1), Canada (CA3,097,204), and Australia (AU2020260379) that protect the technology resulting from the work reported in this manuscript.

## Figures and Tables

**Table 1:** Differential molecular composition of peanuts under raw and thermally processed conditions using two complementary ^1^H NMR techniques. (+) Symbols indicate the presence of each molecule and the number of symbols represent the relative amounts detected. (–) Symbol indicates undetected levels.

Peanut Form	1H NMR Technique	Molecules Detected			Signatures across Conditions (Raw, Roasted, Autoclaved)
		Lipids (Triglycerides)	Sugars (Sucrose, Glucose)	Proteins (Peptides, Amino Acids)	
Whole, intact peanut	HR-MAS	+++	+	–	No major change
Defatted peanut flour	HR-MAS	+	+++	+/–	No major change
Peanut-soaked solution	Solution	–	+++	++	Distinct for autoclave (broad peaks)

## Data Availability

Data are contained within the article.
